# Maskit’s Mathematical Contributions: The Smoothing Operator and DAAP Measures

**DOI:** 10.1007/s10936-025-10158-0

**Published:** 2025-07-08

**Authors:** Perry Susskind

**Affiliations:** Department of Mathematics & Statistics, Connecticut College, 270 Mohegan Avenue, New London, CT 06320 USA

**Keywords:** Smoothing operator, Weighting function, DAAP, Multiple code theory

## Abstract

An elementary explanation of Bernard Maskit’s mathematical contributions to multiple code theory. The presentation is limited to an exploration of Maskit’s use of mathematical smoothing in defining a number of measures that, given a narrative, provide information about the emotional experiences that are conveyed by that narrative.

## Introduction

Below we provide an elementary explanation of Bernard Maskit’s mathematical contributions to *multiple code theory* (Bucci, 2021), and the problem of understanding in psycholinguistic terms the process of conveying emotional (nonverbal) experiences using language. Working with his wife and co-theorist, Wilma Bucci, Maskit made numerous and extensive contributions in this area; his mathematical approach and introduction of the mathematical technique called *smoothing* provided a means for deriving a number of important measures that led to substantial progress in the theory. At the simplest level, the use of smoothing allowed for the analysis of a text whereby, instead of considering the constituent words individually, the individual words are evaluated by also taking into account the values for nearby words of the text. Thus, Maskit’s innovation provides an almost deictic aspect in evaluating each word of a text. More specifically, this mathematical work was brought to bear in implementations of the *Discourse Attributes Analysis Program* (DAAP) and in defining various measures such as the *Mean High WRAD*, the *High WRAD Proportion*, and the *covariations* of several measures (Maskit, 2021b, a). These measures all make use of a *smoothing operator* that Maskit defines, found in Maskit (2014, 2012, 2021a), and the appendix of Bucci et al. (2015).^1^ The DAAP makes use of a dictionary which assigns to each of the listed words a numerical value. There are several dictionaries in use; each is one of two sorts: In a *weighted* dictionary, a numerical value is assigned to each word in the dictionary. For words that are not in the dictionary a numerical value corresponding to a “neutral value” is used. In a *non-weighted* dictionary, included words are given one numerical value; words that are not included in the dictionary are given another value, which may also correspond to a “neutral value”; see Maskit (2014). Thus, for any dictionary that is used by the DAAP, all words, whether in the given dictionary or not, are assigned numerical values. We shall later define a weighting system that is applied to the dictionary values of the words of a text that relies, in each instance, upon the position of the word within the text. In order to avoid confusion, following Maskit (2014), we shall refer to the numerical values that are assigned to words in a dictionary as “dictionary values,” or if a particular dictionary such as the WRAD is in use, the “WRAD value.” The weights that arise from the position of a word in a given text shall be referred to as “weights.”

The Weighted Referential Activity Dictionary (WRAD), and the Weighted Reflecting Reorganizing List (WRRL) are principal among the weighted dictionaries in use. For each dictionary the assignment of numerical values are instrumental in defining measures of emotional engagement, or other psychological variables (Maskit et al., 2024). In the DAAP, with a particular dictionary, Maskit’s smoothing operator is applied to the numerical stream associated to a particular text (either a list of numbers, or a function that assigns to each moment over a period of time, a numerical value) in order to produce a measure of the emotional or psychological variables conveyed by the text. The presentation herein follows the outline of both *DAAP Math I: Word Count Base* (Maskit, 2014) and *DAAP math II: Variable Time Basis* (Maskit, 2012), and particularly follows the former reasonably closely, with the focus being on an elementary presentation of a number of mathematical definitions and constructions that Maskit employs, rather than on explication of the use of the mathematics, beyond the definitions, in multiple code theory. While, mathematically speaking, there is nothing new (except perhaps for comparisons with the raised cosine distribution), the hope is that the elementary nature of the exposition will be helpful. (We suggest that those who wish to learn about Maskit’s mathematical contributions to the DAAP without paying attention to all of the mathematical details may wish to read what follows without undue attention to the footnotes numbered 13 and above. Those who are more mathematically curious may wish to also consider those footnotes as well. The appendix, which includes details about the use of the integral in smoothing, may also be consulted.)

In attempting to understand emotional, sensory and somatic experiences as conveyed in a narrative form (usually transcribed from a psychotherapy session), one basic obstruction is that these experiences are perceived as occurring continuously in time, but some constituents of a narrative – words – are discrete. At a more technical level, there are a number of temporal aspects of a verbal narrative that are continuous in nature, while others are discrete. For example, a narrative may be broken into segments that begin and end at specific moments in time that lie along a continuum (Maskit, 2012). Other measures of a spoken narrative include acoustic measures such as pitch, standard deviation of pitch, and power. The “PRAAT” measure produces averages of these measures (Maskit, 2012). We will not consider these measures in what follows.

**Fig. 1:**
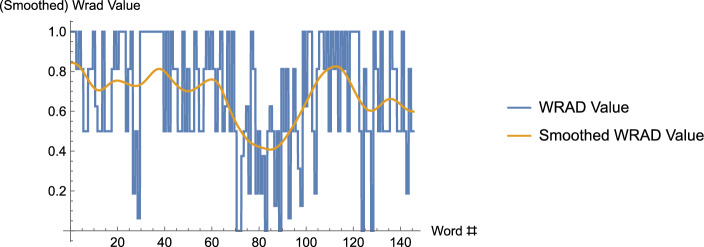
The graph, consisting of vertical and horizontal segments, is the WRAD data. The horizontal segments represent the WRAD values for each of the 145 words of the text – the song, Long Black Veil, by Lefty Frizzel; the (curvy) curve that appears is that data smoothed. Note, in the curve that results from smoothing, one can see the “trend" of the data far more easily

Of course, as observed above, the words of a narrative are discrete entities (Maskit, 2014). Maskit’s use of a smoothing operator allowed a number of fundamentally discrete entities to be directly compared with others that are either discrete or continuous in nature in a number of applications. In addition, consider discrete or continuous values that are highly variable, from one to the next in the discrete case (for example, by employing a *dictionary* that assigns numerical values to each word, contiguous spoken words may be assigned numerical values), or which oscillate rapidly in the continuous case.^2^ See Fig. 1 below where, by using the WRAD, a text is converted to a discrete but highly oscillatory stream of numerical values that is then smoothed. We shall expend considerable effort in this paper in defining how this smoothing is accomplished. As another example, imagine that one wishes to plot the price of a given stock over time. The stock price at a particular time and day may not have terribly much meaning, but the average price of the stock within a window centered at a particular time and day, may provide a far more meaningful indication of the stock price; moreover, looking at this “smoothed"price over time may provide far more information about the trend than the bouncy individual prices that are subject to significant fluctuations from moment to moment. Streams of values arising from data are often highly oscillatory and thus are difficult to compare directly with other such streams of values. For example, see Maskit (2021b), and in that paper, compare figure 1, which contains unsmoothed data that arose from assigning numerical values to each word occurring in a narrative – by making use of the WRRL dictionary – with the smoothed version appearing in figure 3. However, when two original data streams, both of which may be highly oscillatory, are smoothed, direct comparisons (including covariation between the two streams of values) may be made. (For instance, perhaps values are assigned using two different dictionaries. See Fig. 6 below). In what follows we describe the three steps used in defining the smoothing operator. After doing so, we describe in a few examples how the smoothing operator may be applied.

Before proceeding, we thank Ara Basmajian, Wilma Bucci, Gabriel Chandler, Charles Hartman and Attà Negri for helpful contributions to this paper.

## The Smoothing Operator

### The Set-Up

**Fig. 2:**
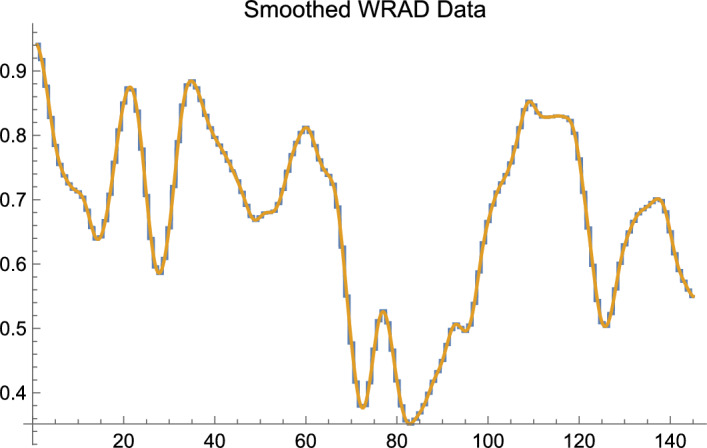
The horizontal axis is Word #; the vertical axis is Smoothed WRAD Data. Smoothing, as in the earlier figure, is of the text of Long Black Veil. The graph consisting of vertical and horizontal components, with the requisite property of “jaggedness,” is the result of smoothing using finite sums to compute the weighted averages that are required to perform smoothing. The smooth curve is the result of smoothing using the integral to compute the required weighted averages. One easily sees that, qualitatively speaking, except for the property of literal smoothness, little is lost by employing the simpler procedure of employing finite sums to compute averages as compared with using the integral to do the same

In order to make the exposition clear and to avoid in our main example using the machinery of calculus, we will work with finite, discrete data of a particular sort; but the construction, as should be obvious once the mathematical work has been described, is far more general. Below, in order to compute averages, and perform smoothing, we shall make use of finite sums in working with finite, discrete data. It is a relatively simple matter, when dealing with finite discrete data, or continuous data, to replace the finite sums,^3^ as Maskit has in certain applications, with the integral from calculus. Almost all discussion herein that employs the integral shall be relegated to the footnotes. We also provide an appendix at the end of this article that provides a short primer on the use of the integral. Figure 2 illustrates that for discrete data there is little lost in making use of finite sums rather than the more powerful integral.

We suppose that we have a text (which usually arises as a narrative from a psychotherapy session) that consists of a number of contiguous words. We also make use of a dictionary; for this set-up we use what is called a *weighted dictionary* that assigns to many words a numerical value.^4^ Thus, for each word in the text, there is an associated number.^5^ Therefore, by associating to each contiguous word of the text the number that appears in the dictionary for that word, for the entire text there is an associated ordered list of contiguous numbers.

### The Window

We now have an ordered list of numbers that is associated to the text that was provided. Given the ordering of words in the text, the associated dictionary values will tend to jump around quite a bit as this list is traversed from left to right. We will attempt to dampen or *smooth* this high variability in two ways that successively increase in usefulness. Our first attempt will be through a simple averaging process. Pick a fixed integer, *m*, that is greater than 1 (and which is smaller than the length of the text^6^). Suppose we are at a particular point in our text. Instead of simply associating the dictionary value for that word, we instead associate the average of the dictionary values for the word itself and the contiguous words that lie within $m-1$ words to the left and $m-1$ words to the right of the given location. The number of words, or corresponding dictionary values, that are considered in the average (weighted or unweighted, see below) is called the *window size*. Note: As the analysis below shows, the choice of the positive integer, *m*, results in a window size of $2m-1.$^7^ For example, suppose *m* is 4 and the text is “*The soul wears out the breast, the heart must pause to breathe, and love itself have rest,*” slightly altered lines from Lord Byron’s poem, *So We’ll Go No More a Roving.*,^8^ Later, as suggested by the earlier figures, we shall use a somewhat longer text, the song, Long Black Veil, by Lefty Frizzel.^9^ Suppose also that we are located at the word, *must,* in the text. Instead of simply taking the numerical value assigned to the word, *must,* from the dictionary (0.1875), since $m-1$ is 3, we instead compute the average of the dictionary values assigned to the words, *breast,* *the,* *heart,* *must,* *pause,* *to,* *breathe,* that is, the average of all values within 3 words to the left and three words to the right of our location within the text. One can see that for the choice of 3 for the value of $m-1,$ the size of the window of values for which the average is computed is 7, 3 words to the left of the chosen word, 3 values to the right, as well as the chosen word itself. It happens that the dictionary WRAD produces the list of values, (1, 0.5, 0.5, 0.9375, 1, 0.5, 1, 0.5, 0.1875, 0.5, 0.625, 0.5, 1, 0.375, 0.5, 0.5, 0.375) for the text we chose. (See the first two columns of Table 1 below.) Thus, in order to produce the simple average of the dictionary values for the 7 words centered at *must*, we compute, $\frac{1}{7} 0.5 + \frac{1}{7} 1+ \frac{1}{7} 0.5 + \frac{1}{7} 0.1875 + \frac{1}{7} 0.5 + \frac{1}{7} 0.625 + \frac{1}{7} 0.5 = 0.54464.$^10^ Thus, where the dictionary value for the word *must* is 0.1875, the average value for the word *must* is 0.54464. (This value appears in the third column of Table 1, on the line containing the word, *must.*)

There are two drawbacks with the process described above: (1) The process breaks down when we are close to the very beginning or the very end of the text. For example, when we are located at the second word of the text, the word, *soul,* there are not 3 words to the left of the second position to use in computing the average. Similarly, if we are located at the word, *have,* there aren’t 3 words to the right of that location to use in computing the average. (Thus, at present, we are unable to fill out all of the entries in Column 3 of Table 1, as the entries on the initial 3, and final 3, lines of Column 3 are not defined.) We deal with this issue in the next section by performing a “foldover operation"or “wraparound operation"on the text data. (2) For *m* of any significant size, we may wish data that lies very close to the chosen location in the text to have more weight, or influence, in the computed average than data that is more remote from the chosen position in the data. That is, the weights given to dictionary values in the average should diminish as we get closer and closer to the boundaries of the window. In the DAAP, the value $m=100$ is often used, providing a corresponding window size of 199. It is advantageous for words clustered near the chosen word at the center of the window to have more weight in the computed average than the words that are more remote from the center. In our example above, the dictionary value of each word is equally weighted with weight $\frac{1}{7}$ in the average, that is, each of the seven dictionary values is multiplied by the corresponding weight, $\frac{1}{7},$ and these products are then added up to produce the average. In a (non-constant) weighted average, the seven weights within the window might be chosen to be $\frac{1}{16}, \frac{1}{8}, \frac{3}{16},\frac{1}{4},\frac{3}{16},\frac{1}{8},\frac{1}{16},$ in that order. (One can check that the weights add up to 1, or 100%, a requirement of any weighting, and that the list is symmetric with respect to the middle entry, with weights decreasing as one tends away from the middle.) As before, the dictionary value of the each word in the window is multiplied by the corresponding weight, and those products are added up to produce the weighted average. In this instance, the weighted average, employing the WRAD, would be $\frac{1}{16}0.5 + \frac{1}{8} 1+ \frac{3}{16} 0.5 + \frac{1}{4} 0.1875 + \frac{3}{16} 0.5 + \frac{1}{8} 0.625 + \frac{1}{16} 0.5 = 0.50000.$ (Note that this value appears in column 4 of Table 1, along the line beginning with the word, *must*. As before, there remains the problem of filling out the values for this weighted average at the beginning three and final three lines of the table.) Keep in mind that the particular weighting, $\frac{1}{16}, \frac{1}{8}, \frac{3}{16},\frac{1}{4},\frac{3}{16},\frac{1}{8},\frac{1}{16},$ was chosen for illustrative purposes only. In the second section below, we describe instead how to judiciously choose a weighting for a given choice of *m*, or correspondingly, a given window size of $2m-1.$ (The use of this latter weighting is illustrated in Column 4 of Table 1, but more on this later).

### The Foldover Operation

Here we deal with the problem of the (weighted) average within a window being undefined near the beginning, or near the end, of the text. The solution is to simply “flip"or “foldover" the text on each side. This pads each end of the text so that there is ample and appropriate data for all original words of text to compute the average. In our example, the foldover on each side of the original text would produce the text (original text in boldface): “*rest, have, itself, love, and, breathe, to, pause, must, heart, the, breast, the, out, wears, soul, The,* ***The, soul, wears, out, the, breast, the, heart, must, pause, to, breathe, and, love, itself, have, rest,*** *rest, have, itself, love, and, breathe, to, pause, must, heart, the, breast, the, out, wears, soul, The.*”^11^^,^ 12^,^ 13 For example, if as above, $m=4,$ and the location in the text is *soul,* each of the numerical dictionary values for the words of text, *soul, The,* ***The,*** *soul*, ***wears, out, the*** is multiplied by the corresponding weight; here the weight values are centered on the second occurrence in the sequence of the word, *soul* (underlined), in the sequence above, and then the terms are added up to produce the weighted average. Thus, using the weights, $\frac{1}{16}, \frac{1}{8}, \frac{3}{16},\frac{1}{4},\frac{3}{16},\frac{1}{8},\frac{1}{16},$ and referring to Table 1 for the dictionary values, we compute the weighted average for the word, *soul,* to be $\frac{1}{16} 0.5 + \frac{1}{8} 1+ \frac{3}{16} 1 + \frac{1}{4} 0.5 + \frac{3}{16} 0.5 + \frac{1}{8} 0.9875 + \frac{1}{16} 1 = 0.74219.$ This entry appears in column 4 of Table 1 in the line corresponding to the word, *soul*. Again, no matter where one is located within the original text, there is ample text data on both sides to compute the weighted average. It should be clear that by employing the foldover operation as we have, given a choice of weights, it is now possible to fill in all of the columns in Table 1, particularly the initial few and final few lines. We have not yet said where the weights that are used for column 5 come from. This will be the topic of the next section. We assume throughout that *m* is chosen so that the window size, $2m-1,$ is (usually significantly) smaller than the number of words in the text.^14^

**Table 1:** Different Weighted Averages of WRAD Values

Text	WRAD Value	Uniform Average	Example Smoothed	Maskit Smoothed
the	1	0.70536	0.74609	0.83296
soul	0.5	0.77679	0.74219	0.66486
wears	0.5	0.77679	0.73828	0.65052
out	0.9375	0.77679	0.76563	0.80786
the	1	0.70536	0.76953	0.83385
breast	0.5	0.66071	0.72266	0.77964
the	1	0.66071	0.67578	0.70729
heart	0.5	0.61607	0.57422	0.54958
must	0.1875	0.54464	0.50000	0.39630
pause	0.5	0.61607	0.52734	0.45043
to	0.625	0.52679	0.54688	0.55672
breathe	0.5	0.52679	0.58203	0.66090
and	1	0.57143	0.61719	0.67206
love	0.375	0.55357	0.56250	0.58201
itself	0.5	0.51786	0.51563	0.48002
have	0.5	0.51786	0.47656	0.45824
rest	0.375	0.44643	0.43750	0.41676

In the column, “Uniform Average,” each entry in the window (of width 7) is given the weight $\frac{1}{7}$; in the column “Example Smoothed,” the WRAD Values are weighted by $\frac{1}{16}, \frac{1}{8},\frac{3}{16},\frac{1}{4},\frac{3}{16},\frac{1}{8},\frac{1}{16}$ respectively, as in the example provided above; in the column labeled “Maskit Smoothed,” the corresponding weights are: 0.0000002, 0.0348412, 0.2644011, 0.4015150, 0.2644011, 0.0348412, 0.0000002. See below for how these weights arise. Note also that in practice, only the weighted average listed in the last column is used

### The Weighting Function

We now consider the problem of choosing a viable set of weights for our situation in which we have discrete data with a finite window size of $2m-1.$ The motivation for these choices will follow. We first define a carefully chosen weighting function that lies along the number line and is centered at 0. In this situation, the non-zero weights are chosen within the window defined by starting $m-1$ units to the left of the point 0, that is at $1-m,$ and ending $m-1$ units to the right of 0, that is at $m-1.$ The weights assigned to the value $-m,$ and to the left of $-m,$ and at $+m,$ and to the right of $+m,$ are all 0. In our tiny example above, where *m* is 4, the weighting function has non-zero values at the points from 3 units to the left of 0 to 3 units to the right of 0, that is, from $-3$ to $+3.$ Values at $-4$ and to the left of $-4$, as well as at $+4$ and to the right of $+4$ are all 0.^15^

Later, in the next section, when computing the weighted average for a given data point, we shall “shift"or “slide"or “offset"the weighting function so that its value at the point 0 lies over the chosen data point. In somewhat less technical language, we will choose an ordered list of exactly $2m-1$ weights (the integers from $-m+1$ to $m-1$), symmetric with respect to the middle weight, with highest value at the middle (which lies over the point 0), decreasing as the list is traversed from the middle to either end. These weights are then slid along the data so that the middle point lies over the chosen data point for which the weighted average is to be computed. This procedure was discussed and employed above where we instead used more arbitrarily chosen weights.

**Fig. 3:**
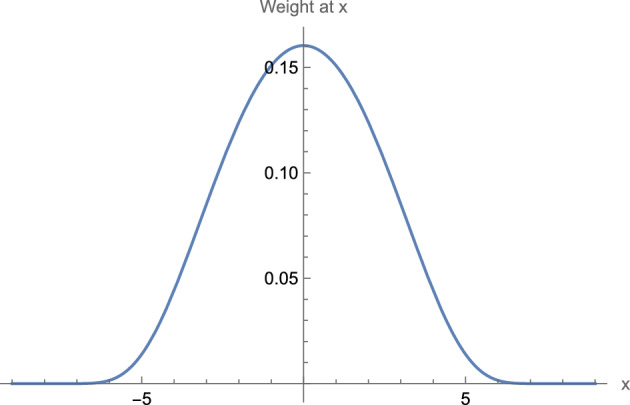
The graph, for a window size of 19 ($m=10$), provides for the value *x* along the horizontal axis, the weight at *x*, read along the vertical axis. Note that the *qualitative* shape of the graph – maximum at $x=0$, symmetric on both sides of $x=0,$ with values that become very, very close to 0 for $|x|>0.63 m$ – will remain the same for all window sizes

One should observe that there are infinitely many choices of weighting functions that satisfy the criteria discussed above and which could be used for this and other applications. Given additional motivations discussed below, Maskit defines a bell-curve-like function that, as discussed above, produces nonzero weights at points from $1-m$ to $m-1$, and weights of 0 outside of this window. That is, the chosen function satisfies the criteria above wherein the weighting function has nonzero weights for positions up to $m-1$ units to the left of 0, and up to $m-1$ units to the right of 0, with weights of 0 elsewhere. Moreover the weighting function is symmetric, has its largest value at the 0 th position, and decreases in both directions.^16^ Maskit calls this the *moving weighted average.* See Fig. 3.

The motivation for the particular choice of weighting function Maskit uses arises from the situation of numerical data that lies along a bi-infinite continuum, that is, for data that is defined for all of the real numbers. Though it is outside of the scope of this paper to discuss them at length, there are often good reasons in this case to choose a weighting function that is a standard “Gaussian,"that is, the weighting function is the standard “bell curve"of mean 0 and standard deviation 1.^17^ The weighting function that Maskit chose for data on a finite interval has many of the properties of the standard bell curve, though it has nonzero values only on the finite (open) interval from $-m$ to *m*.^18^^,^^19^^,^^20^

### The Weighted Average and the Smoothing Operator

We return to the situation defined earlier, in which there is a finite, discrete set of numerical data that has possibly come about by associating each word in a text with a numerical value that resides in a dictionary. As above, we have also chosen a positive integer *m* that provides a window size of $2m-1.$ In order to compute the weights centered at a given point in our data, so as to arrive at a weighted average, much as we did before (but with a more judicious choice of weights), the bell-curve-like weighting function discussed (for this value of *m*) in the previous subsection is shifted, or slid, so that the weight lying over 0, (where the bell curve is highest) is placed over the given point in the data, with the rest of the weights decreasing in a symmetric fashion as one tends away from the chosen point. Thus, the weighted average for a particular point in the text is then obtained, as in the example in the previous section, by matching the dictionary values within the given window centered at the particular point in the text, with the weights that occur when the weighting function is placed so that the 0 position lies over the chosen point in the text. Finally, the *smoothing function* or *smoothing operator* is defined as precisely this weighted average.^21^ For our earlier example, the values in the final column of Table 1, above, are computed in exactly this fashion.

As another example, in the instance of the smoothing of continuous data using the standard Gaussian, the standard bell curve, in a similar fashion to the operation above, is slid along the number line until the point where the bell curve is highest lies over the given data point. The corresponding values provided by the bell curve are then used as weights for the corresponding data points.^22^

**Fig. 4:**
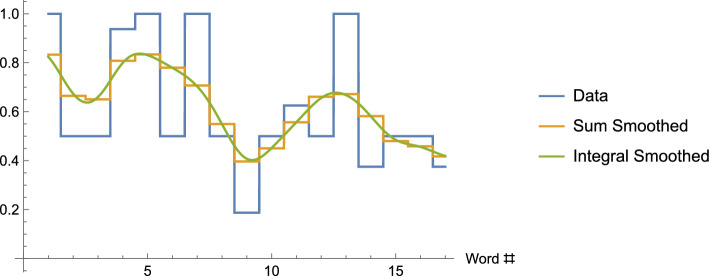
The graph shows the original data for our example, as well as the sum-smoothed data (Column 5 of Table 1), and integral-smoothed data

Finally, if we have continuously defined data and wish to compute a smoothing function at the point *x* where the nonzero weights occur only in the interval from $x-m$ to $x+m,$ Maskit uses a bell-curve-like weight function similar to the one that began this section but modified for continuous data.^23^ See Fig. 4.

**Fig. 5:**
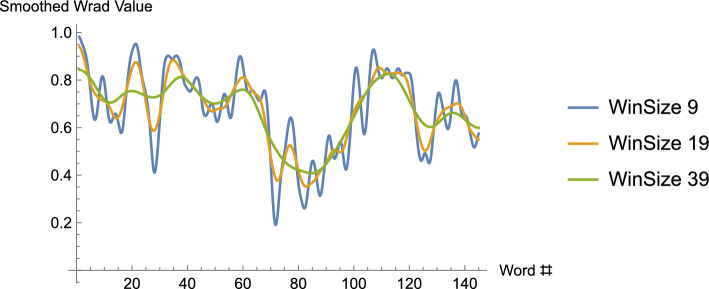
The graph, again using the WRAD values from the song, Long Black Veil, is smoothed using the integral – though we’ve seen that this only means the smoothed curves are “curvy” rather than consisting of horizontal and vertical lines – for three different window sizes, 9, 19 and 39

One issue remains. Above, we observed that the window size for the smoothing function relies on the choice of an integer *m* (and is $2m-1$ for a given choice of *m*). What is the effect of choosing different window sizes? The answer is that for increasing window sizes the data is increasingly smoothed. Indeed, as the window size is chosen to be closer and closer to the length of the data, the smoothing would result in an increasingly un-wavy-looking curve). (See Fig. 5.)

## Applications

The description of Maskit’s smoothing operator is complete. It only remains to list briefly some of the applications.

### Means

It is an elementary fact for either a finite list of discrete numerical values, or for a set of continuously defined values on an interval of real numbers, that the mean value of (all of) the data is equal to the mean value of (all of) the smoothed data. Thus Maskit’s smoothing leaves the mean values of data unchanged, and whatever meaning this measure is taken to have is retained for smoothed data.

### Derived Measures

Consider the DAAP that makes use of the dictionary, WRAD. (These derived measures can be defined in precisely the same way for other dictionaries.) The “neutral value"of the WRAD is 0.5, and, given a text, it is of interest to consider separately the words that have smoothed values that are bigger than the neutral value, and the words whose smoothed values are less than or equal to the neutral value. We partition the words of the text into two collections. Let *H* be the collection of words that have smoothed values that are bigger than the neutral value, L the collection of words with smoothed values that are less than or equal to the neutral value. For a word that appears more than once in the text, it also appears the same number of times in the collection, *H*, or *L*. (For the sample text we chose to work with earlier, by using Table 1 we see that $H=\{the, soul, wears, out, the, breast, the, heart, to, breathe, and, love\}$; $L=\{must, pause, itself, have, rest\}$.) Suppose also that |*H*| is the number of words in the collection *H*, that is, |*H*| is the number of words of the text with smoothed values that are greater than the neutral value, and similarly, |*L*| is the number of words that have smoothed values that are less than or equal to the neutral value.^24^ Suppose *N* is the total number of words in the text. Then, $|H|+|L|=N.$ (For our sample text we have, $|L|=5$, and $|H|=12$, $N=17.$) The *High WRAD Proportion*, or *HWP*, is simply the proportion of smoothed words in the text that have larger than neutral value; clearly, *HWP* is the number of words of text that have smoothed value bigger than the neutral value (in this case, 0.5), divided by the total number of words of text, that is, $HWP = |H|/N.$ (For our example, $HWP = 12/17 = 0.70588.$) We define the *Mean High WRAD*, denoted *MHW*, to be 0 if no words of the text have smoothed values larger than the neutral value, that is if $|H|=0$, and otherwise, *MHW* is the average smoothed value of all the words of *H* minus the neutral value, 0.5.^25^^,^ 26 (Using Table 1, we may not so easily compute for our example that $MHW = (0.83296+0.66486+0.65052+0.80786+0.83385+0.77964+0.70729+0.54958+0.55672+0.66090+0.67206+0.58201)/12- 0.5 = 0.191521.$)

Keep in mind that since membership in the collection, *H*, relies, as do the smoothed values for the text that is being analyzed, on the window size of the smoothing function, it is possible that *H*, *HWP*, and *MHW* may all possibly change somewhat with a change in window size. This will occur if the smoothed values of one or more individual words cross the neutral value, 0.5, when the window size is changed.

### Covariation

Suppose there are two dictionaries in use for a given text. Then, the values assigned to the constituent words, as well as the smoothed values, will be different, depending upon which dictionary is in use. How shall these be compared? Suppose that two dictionaries are in use, Dictionary 1 and Dictionary 2. Suppose also that for a given word *w* of a text consisting of *N* words, $S_1(w)$ is the smoothed value of *w* when Dictionary 1 is used in the DAAP and $S_2(w)$ is the smoothed value of *w* when Dictionary 2 is used. We would like to compare the relative correlation between the values that arise using the two different dictionaries. Suppose the two dictionaries have neutral values, $n_1$ and $n_2$, respectively; these may be different or the same.

Typically, given numerical data, one is often interested in two measures: the *mean* – this is just the usual average – and some measure of *dispersion* about the mean. (Usually, the latter is the *variance* or the *standard deviation*.^27^) Given a text, for each word of the text, consider the square of the difference of the smoothed value and the neutral value, that is, for a word *w*, consider the quantity, $(S_1(w)-n_1)^2$. (Here we are using Dictionary 1.) We define the *skewed variation*, $V_1$, to be the square root of the sum of these values over the entire text.^28^^,^ 29$V_2$ is defined similarly, using the function $S_2(w)$. In contrast with the typical situation in which the dispersion about the mean is measured, here we are measuring dispersion about the neutral value instead. (Of course, if the two values agree, the skewed variation times the constant, $\sqrt{\frac{1}{N}}$, is just the usual standard deviation.). We are now able to define the covariation. Given a word, *w*, and smoothing functions, $S_1,$ and $S_2,$ for dictionaries 1 and 2, respectively, consider the product, $(S_1(w)-n_1)(S_2(w)-n_2)$. Let *V* be the sum of all of these products, over all of the words of the text. If both skewed variations are nonzero, then the *covariation*, *C*, is given by $C=\frac{V}{V_1 V_2}.$^30^

**Fig. 6:**
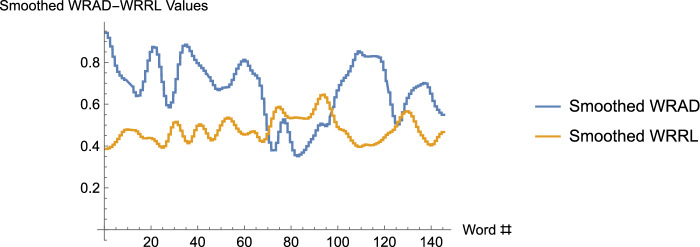
In these graphs, the two curves (which really consist of 145 discrete data points each) represent the sum-smoothed WRAD values, and the sum-smoothed WRRL values, for the song, Long Black Veil, with window size 19. One observes that for substantial pieces of the curves, the values on the WRAD curve lie above the neutral value, 0.5, while the values along the WRRL curve lie below the neutral value. Indeed, the mean value along the smoothed WRAD curve is 0.671121, while the mean value along the smoothed WRRL curve is 0.47765. One might guess, then, that the covariation is negative. A calculation shows that it is $-$0.693209, a value quite close to the Pearson correlation coefficient of $-$0.703238

Note that if dictionary values for dictionaries 1 and 2 were the same for the entire text, i.e., $S_1(w)=S_2(w)$ for all words *w* of the text, then we would have $V_1=V_2$, $V=V_1^2=V_2^2$, and $C=1.$ Generally, it can easily be shown that the covariation lies between −1 and +1 (inclusive). Indeed, if the neutral values of the dictionaries were equal to the respective means, in that case, the covariation, *C*, would be computationally identical to the Pearson correlation coefficient. The latter is used to measure the extent to which there is a linear relationship between two lists of data, with +1 corresponding to a perfect positive linear correlation between the two sets of ordered data. The covariation that we’ve just defined functions in a similar fashion.

Unraveling further the meaning of this, consider again the product, $(S_1(w)-n_1)(S_2(w)-n_2)$. Suppose that $(S_2(w)-n_2)$ tends to be positive whenever $(S_1(w)-n_1)$ is positive, and vice versa, (and moreover, $(S_2(w)-n_2)$ tends to be negative whenever $(S_1(w)-n_1)$ is negative, and vice versa). In such a case, note then that *V*, the sum of these products, will be positive, and that the dictionary values of Dictionary 1 and Dictionary 2 will have positive covariation for a given text. If, on the other hand, $(S_2(w)-n_2)$ tends to be negative when $(S_1(w)-n_1)$ is positive, etc., then the covariation will tend to be negative. $C=\frac{V}{V_1V_2}$ is a “normalized"version of *V*, and indicates the degree to which (in this example) one dictionary has values that are correlated to the values of another dictionary, relative to their respective neutral values, for a given text. See Fig. 6. Keep in mind once again that since smoothing functions $S_1(w)$ and $S_2(w)$ are in use, the actual values for the covariation depend somewhat on the window size that is chosen.

## Final Remarks

Although we have confined our focus on the mathematical constructions Maskit used in the DAAP, perhaps one should also mention that his work on the DAAP was/is by no means complete. Maskit and others were/are involved in continuing work and research on a refinement to the DAAP that we mentioned in passing at the beginning of this paper. This refinement is called the *Time DAAP* or *TDAAP*. This enhanced program, not only notes, and makes use of, which words were spoken in a narrative, but also marks or records the entirety of: (i) the precise moments at which each spoken word in a narrative begins and ends, (ii) the precise moments at which gaps in speech begin and end. Thus, along with summary data that is derived from the original DAAP (such as Mean Dictionary Values, Mean High Dictionary Values such as Mean High WRAD, High Dictionary Proportions such as High WRAD Proportion, and Covariations between different dictionary values), as well as in the future possibly matching linguistic data with voice data such as loudness and pitch – see Maskit et al. (2025) – the promise of this refinement is that analysis of the data gathered will allow for enhanced interpretation of a number of psychological variables that may be apprehended through spoken language.

Finally, after having been entangled in the mathematical details of how, in order to carry forward multiple code theory, smoothing was implemented by Maskit in the DAAP, it is clear, taking a step back, that the effort has resulted in two related outcomes that function sympathetically in this instance: (1) Given a narrative, the DAAP, and associated measures, can turn the experience of an emotional interaction into a process that can be quantified. (2) From a converse perspective, analogously to a physician interpreting an EKG, the graphs and measures can be used by a viewer (a researcher or a therapist) to enable interpretations of the emotional contents of the narrative that may not have been otherwise revealed. It is, indeed, particularly satisfying when the implementation of a highly mathematical process provides consequences of both of these types. Maskit’s introduction of smoothing and work on the DAAP has, quite ingeniously, resulted in both of these attributes. One expects that continuing Maskit’s work on the *TDAAP* will result in further improvements in both of these attributes.

## Appendix: Finite Sums and the Integral

If we were using the mathematics needed to perform Maskit’s smoothing in a completely unrestrained way in this paper, we would be using the integral from calculus in many places. The following is an informal (non-rigorous) discussion of the integral in the context of computing weighted averages.

Smoothing, simply put, is performed by computing a weighted average value of either a finite or an infinite collection of values. In the former case only a finite sum is required to compute this weighted average. In the latter case, the integral is used. Often, even when the data derives from using using the dictionary values from a given dictionary for a text consisting of finitely many words, the related function that is then smoothed may be considered to consist of an infinite collection of values defined over an interval of real numbers. Thus, in such cases the integral may be used, although in this paper we often have instead approximated such averages by employing a finite sum of a finite collection of values instead of using the integral.

To explain this further, suppose we are given a function, $y=f(x),$ defined on an interval of real numbers. Such a function assigns a numerical value – given a number *x*, the associated number is called *f*(*x*) – for each number *x* that lies between two numbers, *a* and *b* where $a<b$. (The values of *f*(*x*) comprise the infinite collection of values we just referred to above.) For simplicity, we will assume that each of the assigned values *f*(*x*) is nonnegative. The graph of this function is a curve in the plane that lies over the horizontal axis, between the values *a* and *b*. (See, for example, Fig. 3, in which is displayed the graph of a function *w*(*x*) defined on the interval of values between -9 and 9.) The *definite integral,* which is denoted by the symbols, $\int _a^b f(x) dx,$ is the area under the curve $y=f(x)$ lying over the interval of values between *a* and *b*. The ability to compute this area is one of the great achievements of calculus.

Back to averages: If one makes use of the integral, one can calculate the (non-weighted) *average value* or the *average height* of the function over an interval by dividing by the area, $\int _a^b f(x)\ dx,$ by the length, $b-a$, of the interval on which *f* was defined. Simply put, if one has variable height, then the average height will be the area of the region, divided by the length of the horizontal base. (If one forms a rectangle with the same length, $b-a$, as we have for the function *f*(*x*), and height, the average height of *f*(*x*) over the interval, then one has constructed a rectangle with the same area as the area under the curve *f*(*x*).) Thus the average value of *f* is $\frac{1}{b-a} \int _a^b f(x)\ dx.$ (By design, the area under the curve in Fig. 3 is 1, that is, $\int _{-9}^9 w(x) dx=1.$ Since the horizontal interval for this function stretches from -9 to 9, and so is of width 18, the average value of the function is $\frac{1}{9-(-9)} \int _{-9}^9 w(x) dx = \frac{1}{18} 1 = \frac{1}{18} \approx 0.0555556$.)

On the other hand, if one does not wish to employ the integral, one might then approximate this average by instead choosing a number, say *n*, of equally spaced numbers, say $a<x_1<x_2<x_3< \cdots <x_n=b$ that divide the interval from *a* to *b* into *n* equally spaced intervals, compute the value of the function *f* for each of these numbers, and then divide by the number *n* of values. Thus, one could say that the average value of *f* is approximately, $\frac{f(x_1)+f(x_2)+\cdots +f(x_n)}{n}$ (or written equivalently using “Sigma notation,"$\frac{1}{n}\Sigma _{k=1}^n f(x_k)$). Put another way, one computes *n* equally spaced test heights for *f*, and then averages them by summing them up and dividing by *n*. Thus,

$$\frac{f(x_1)+f(x_2)+\cdots +f(x_n)}{n}\approx \frac{1}{b-a} \int _a^b f(x)\ dx.$$

(For example, if the formula for computing values of *w*(*x*) is available, and we approximate the average value of the function *w*(*x*) on the interval from -9 to 9 by choosing 6 equally spaced points, we may compute, $\frac{w(-6)+w(-3)+w(0)+w(3)+w(6)+w(9)}{6} = \frac{0.00154783+0.0851955 +0.160877+0.0851955+0.00154783+0}{6}=0.0556427,$ a quite close approximation to the average value obtained by computing the integral.) The finite sums we refer to in this paper are often used to approximate the various averages needed to perform smoothing, in lieu of instead using the integral.

Though it is entirely beyond the aims of this paper to explore calculus, it is germane to mention that the integral in calculus is defined, in essence, by allowing the approximation to the area, $(f(x_1)+f(x_2)+\cdots +f(x_n))(\frac{b-a}{n})\approx \int _a^b f(x)\ dx,$ (i.e., the expression on the left of the symbol, $\approx$), to become precise by employing a “limiting process” in which the number *n* of values is allowed to become larger and larger without bound.

Computing weighted averages requires a few enhancements to this basic picture, but is essentially similar. First, the situation of finitely many values: Rewrite the approximation, $\frac{ f(x_1)+f(x_2)+\cdots +f(x_n)}{n}$ in the form, $\frac{1}{n}f(x_1)+\frac{1}{n} f(x_2)+\cdots +\frac{1}{n}f(x_n)$. We see that this average of the *n* values, $f(x_1), f(x_2), \cdots f(x_n)$ is *weighted* with equal weights, each with the value $\frac{1}{n}$. Note also that the *n* identical weights, $\frac{1}{n}$ add up to 1. In order to weight the values differently, which is required above in order to perform smoothing, instead consider *n* possibly different weights, $w_1, w_2, \cdots w_n$ that also add up to 1, i.e., $w_1+w_2+\cdots +w_n = 1.$ We write the weighted average of the values, $f(x_1), f(x_2), \cdots f(x_n),$ with weights $w_1, w_2, \cdots w_n$, as $w_1 f(x_1)+w_2 f(x_2)+\cdots +w_nf(x_n)$. (This is the process we carried out in the body of the paper.)

For the case of a weighted average of infinitely many values, suppose we wish to perform a weighted average of the function *f*(*x*) over the interval of points from *a* to *b*. Suppose also that we now have available a *weighting function* *w*(*x*) defined over the same interval. (Just as the finitely many weights $w_1, w_2, \cdots w_n$ must add up to 1 in order to perform a weighted average, $w_1 f(x_1)+w_2 f(x_2)+\cdots +w_nf(x_n)$, of the *n* values, $f(x_1), f(x_2), \cdots f(x_n)$, for *w*(*x*) to be a weighting function defined on the interval from *a* to *b* requires $w(x) \ge 0$ and $\int _a^b w(x)dx=1$.) Each of the infinitely many values of *f*(*x*) is weighted with the value *w*(*x*), that is, we compute the product, *w*(*x*)*f*(*x*), for each value of *x* for x that lies between *a* and *b*.. The weighted average we seek is the integral, $\int _a^b w(x)f(x) dx.$ As before, we may wish to approximate this integral by using finite sums, but we will not duplicate this work in this slightly different setting. Finally, we remark that the weighting function that Maskit used was defined and nonzero over a fixed interval whose window size relied upon the parameter *m*, as in footnotes 21 and 23, but this weighting and corresponding weighted average was computed repeatedly over different intervals of values, each centered at dictionary values over all the words of a narrative. This little trick required the use of the *convolution* integral as defined in the same footnotes.

## Footnotes

1 Bernie would be, and was, the first to suggest that *smoothing* is a standard mathematical technique. We observe, nevertheless, that it took significant insight, resulting in a breakthrough of sorts, to realize that employing the technique of smoothing in this area of psycholinguistics could surmount a number of obstructions in applying the theory. Moreover, the particular weighting function that was used – a distribution related to the standard Gaussian – in the construction of the particular smoothing function that was chosen, does not appear to be standard; see (Prabhu, 2014).

2 In the continuous case, in signal processing, the smoothing of highly variable data is often referred to as applying a *low-pass filter.*

3 Let *n* be a positive integer. Given the graph of a nonnegative function over a specific interval, one can approximate the area under the function, and above the horizontal axis of the graph, over the given interval, with a sum having *n* terms. When this sum is divided by the length of the interval that was chosen, one obtains an estimate of the average value of the function over the chosen interval. These are the “finite sums” referred to in the text above. This sum becomes an increasingly accurate estimate of this area, or when divided by the length of the interval, the average value of the function, as the integer *n* is allowed to get larger and larger. By employing a limiting process that allows *n* to increase without bound, the (Riemann) integral from calculus allows one to compute this area, or average value, precisely.

4 Several dictionaries are in use for the DAAP. The numbers assigned to words in a given dictionary are arrived at according to the Referential Process(RP) described in Maskit (2021b). The size of assigned numbers correspond to differing levels of whatever psychological variable is being measured; there also is a *neutral value* assigned to words that do not appear in the dictionary. For our purposes, we need not concentrate on this aspect of the theory.

5 For words in the text that do not appear in the dictionary we assign an agreed upon *neutral value.*

6 The upper bound on the size of *m* may be removed if a slightly more general procedure is used to appropriately pad the data, though there is usually little reason to choose a value of *m* that approaches or exceeds the length of the text. See also, footnote 13.

7 Following the convention in Maskit’s papers, when *m* is chosen, then, for a given location in the text, we look at the *window* of words (or their corresponding dictionary values) consisting of $m-1$ words to the left of the given location, through $m-1$ words to the right of the given location. The $m-1$ words to the left, plus the 1 word at the chosen location itself, plus the $m-1$ words to the right, produce a window size of $(m-1)+1+(m-1) = 2\,m-1$ words. Thus, choosing *m* produces a window size of $2m-1.$

8 Keep in mind throughout that using this short text, and small *window size* provide a “toy"example. That is, the typical length of text analyzed is generally much longer, and the window size used in the smoothing operation is generally considerably bigger. In the DAAP a window size of 199 is typical. The hope is that performing computations with a small example will make clear how smoothing works.

9 Long Black Veil.: Ten years ago, on a cold, dark night / There was someone killed beneath the town hall light / There were few at the scene, but they all did agree / That the man who ran looked a lot like me / She walks these hills in a long black veil / She visits my grave while the night winds wail / Nobody knows, no and nobody sees / Nobody knows but me / The judge said Son, what is your alibi / If you were somewhere else, then you do not have to die / I spoke not a word though it meant my life / For I had been in the arms of my best friend’s wife / The scaffold was high and eternity near / She stood in the crowd and shed not a tear / But sometimes at night when the cold wind moans / In a long black veil she cries over my bones.

10 Note that $$\frac{0.5+1+0.5+0.1875+0.5+0.625+0.5}{7}= \frac{1}{7} 0.5 + \frac{1}{7} 1+ \frac{1}{7} 0.5 + \frac{1}{7} 0.1875 + \frac{1}{7} 0.5 + \frac{1}{7} 0.625 + \frac{1}{7} 0.5 = 0.54464.$$

11 One could have a robust discussion about why the first and last words at the beginning and end of the original text are repeated, but we will not have the discussion here.

12 If one wished, one could first produce the dictionary values for the given text and then, equivalently, perform the foldover process with the numerical data.

13 Maskit did something more sophisticated than we indicate here by, in essence, performing the foldovers ad infinitum so that there was a bi-infinite sequence of values produced. There is theoretical value in doing so, especially when discussing the continuous version of this discrete process, but what we describe above, assuming a window size that is not too large compared with the length of the text, is adequate for the purpose of defining the smoothing operator.

14 If a given text consists of *N* words, and the dictionary value of the word at position *j* of the text is *R*(*j*), for any value of *j* between 1 and *N*, inclusive, then we may extend the function *R* on the right, using the foldover operation, by defining $\tilde{R}(j) = R(j)$ for $j=1, \cdots , N,$ and $\tilde{R}(j)=R(N+1-j)$ for $j=N+1,\cdots ,2N.$ The foldover operation on the left extends the function by defining $\tilde{R}(j)=R(1-j),$ for $j=-N+1,\dots , 0.$ Equivalently, one may complete the foldover extension on the right and then insist that the extended function $\tilde{R}$ is defined for all integers by insisting that it must be periodic, of period 2*N*.

15 In some circumstances, particularly when we elect to use the integral, the weighting function is defined in exactly the same way, but is allowed to take on values for all real numbers.

16 Though some will not be concerned with this level of mathematical detail, for those who are curious, the weighting function described (for a given value of *m*) is:

$$W(x) = \frac{e^{-2\,m^2\frac{m^2+x^2}{(m^2-x^2)^2}}}{K}$$

where

$$K=\Sigma _{j=1-m}^{m-1} e^{-2\,m^2\frac{m^2+j^2}{(m^2-j^2)^2}}$$

for integer values of *x* lying between $1-m$ and $m-1$ inclusive, and $W(x)=0$ for numbers *x* outside of this interval.

17 In this case the weighting function is $W(x)=\frac{1}{\sqrt{2 \pi }}e^{-\frac{1}{2}x^2}$ for $-\infty< x < \infty .$

18 If the situation calls for the analysis of continuous data for which a weighted average is to be computed, with weights that are nonzero only on a finite interval, $(-m,m),$ the weighting function may be expressed as

$$W(x)=\frac{e^{-2m^2\frac{m^2+x^2}{(m^2-x^2)^2}}}{K}$$

where

$$K=\int _{-m}^{m} e^{-2m^2\frac{m^2+t^2}{(m^2-t^2)^2}}dt$$

for values of *x* that lie in the interval $(-m,m),$ and $W(x)=0$ for numbers *x* outside of this interval. One should observe that *W*(*x*) is $C^{\infty }$ or *smooth.* For a weighting function that is to be applied on a bounded interval, there are good reasons to have the weighting function decay to 0 in a smooth fashion. This results in smoothed data that also is reasonably smooth. For this reason, the weighting function *W*(*x*) may be a better choice than the more common,* truncated normal distribution.*

19 It is also germane to mention that the distribution that Maskit chose is very similar in its values to the *raised cosine distribution* which is given by $W(x)=\frac{1}{2\,s} [1+\cos (\frac{x}{s} \pi )]=\frac{1}{s} hvc(\frac{x}{s} \pi )$ for $-s \le x \le s$ and 0 otherwise (where *hvc* is the “haversine" function.) Indeed, by adjusting the parameters *m* and *s*, where *s* is approximately 0.63*m*, the two distributions may be brought into almost uncanny agreement. Maskit’s distribution has the advantage of being $C^\infty$ for all values; the raised cosine distribution is merely $C^1$ at *s*.

20 We mentioned above that there are a multitude of different distributions/weightings that one might choose for different applications, and even different weighings for a given application. If Maskit had made another good, but different, choice, the numbers obtained for the smoothing function would have changed somewhat, but the overall theory and value of the DAAP would remain essentially the same (Prabhu, 2014).

21 For the mathematically curious, the smoothing function, *S*(*n*), is the *convolution* of weight function *W*(*x*) with $\tilde{R}(n)$, where $\tilde{R}(n)$ is the dictionary value of the $n^{th}$ word within the (padded) text. Thus, $S(n)=\Sigma _{j=1-m}^{m-1}W(j)\tilde{R}(n+j).$

22 This is the *convolution* of the weighing function, $W(x)=\frac{1}{\sqrt{2 \pi }}e^{-\frac{1}{2}x^2}$, given by the standard bell curve, and the data function, $\tilde{R}(x),$ whence $S(x)=\int _{-\infty }^{\infty } W(t)\tilde{R}(x+t)dt.$

23 As discussed in an earlier footnote, the weighting function may be expressed as

$$W(x)=\frac{e^{-2m^2\frac{m^2+x^2}{(m^2-x^2)^2}}}{K}$$

where

$$K=\int _{-m}^{m} e^{-2m^2\frac{m^2+t^2}{(m^2-t^2)^2}}dt$$

for values of *x* that lie in the interval $(-m,m),$ and $W(x)=0$ for numbers *x* outside of this interval. The smoothing function is then given by the convolution, $S(x)=\int _{-\infty }^{\infty } W(t)\tilde{R}(x+t)dt= \int _{-m}^{m} W(t)\tilde{R}(x+t)dt.$

24 Although the notation is identical, |*H*| is, as defined, a count of the number of words in a collection, not the absolute value.

25 As a formula we have, $MHW= \frac{1}{|H|} \Sigma _{j \in H} S(j) - 0.5,$ where *S*(*j*) is the smoothed value of word *j*.

26 Also note that many of these measures, especially continuous ones, are computed by evaluating integrals. The sums that appear in the foregoing may be regarded as very accurate approximations to these integrals.

27 Given data values $x_j,$ $j=1, \cdots ,N,$ the (population) variance is given by $\sigma ^2=(1/N)\Sigma _{j=1}^N (x_j-\mu )^2$, where $\mu$ is the mean. The standard deviation, $\sigma ,$ is the square root of the variance.

28 This is just, $V_1=\sqrt{\Sigma (S_1(w)-n_1)^2}$ where the sum is taken over all of the words of the text. If $n_1$ happens to be the mean of the data, then $\sigma = \sqrt{\frac{1}{N}}\ V_1$, where $\sigma$ is the standard deviation.

29 Some may be perplexed by the appearance of the square root in the formulas for the standard deviation and skewed variance. Other than observing that the square root restores the units to that of a distance, rather than a squared distance, it is perhaps best to suggest that one seek an elementary statistics text for further explication.

30 That is, $C=\frac{1}{V_1V_2}\Sigma (S_1(w)-n_1)(S_2(w)-n_2)$, where the sum runs over all of the words of the text.

## References

[CR1] Bucci, W. (2021). Overview of the referential process: The operation of language within and between people. *Journal of Psycholinguistic Research,**50*, 3–15. 33566313 10.1007/s10936-021-09759-2

[CR4] Bucci, W. & Maskit, B. (2006). A weighted dictionary for referential activity. J. G. Shanahan, Y. Qu, & J. Wiebe (Eds), Computing attitude and affect in text: Theory and applications (49-60). Springer. 10.1007/1-4020-4102-0.

[CR5] Bucci, W., Murphy, S., & Maskit, B. (2015). A computer program for tracking the evolution of a psychotherapy treatment. Proceedings of the 2nd workshop on computational linguistics and clinical psychology: From linguistic signal to clinical reality (134-145). Denver, Colorado: Association for Computational Linguistics.

[CR6] Maskit, B. (2012). DAAP math II: Variable time basis. https://figshare.com/articles/journal_contributionDAAP_using_Time_as_Independent_Variable_Technical_Aspects/947741.

[CR7] Maskit, B. (2014). DAAP math I: Word count base. https://figshare.com/articles/journal_contribution/DAAPMath/928469.

[CR8] Maskit, B. (2021a). The DAAP technical manual [Computer software manual]. https://figshare.com/articles/online_resource/The_DAAP_Technical_Manual_pdf/14312096

[CR2] Maskit, B. (2021b). Overview of computer measures of the referential process. *Journal of Psycholinguistic Research,**50*(4), 29–49. 33464426 10.1007/s10936-021-09761-8

[CR3] Maskit, B., & Bucci, W. (2025). A journey through the land of DAAP: Including visits to weighted dictionaries, smoothing, covariations, and the effects of word order, with connections to psychology, psycholinguistics, mathematics and statistics, and ending at time-based DAAP (TDAAP). *Journal of Psycholinguistic Research,**54*(3), 1–7. 10.1007/s10936-025-10145-5 PMC12062042 40338374

[CR9] Maskit, B., Bucci, W. & Murphy, S. (2024). Tracking the ebb and flow of psycholinguistic variables: The discourse attributes analysis program.

[CR10] Prabhu, K. (2014). Window functions and their applications in signal processing. CRC Press. ISBN 978-14665-1583-3

