# Correction: Maskit’s Mathematical Contributions: The Smoothing Operator and DAAP Measures

**DOI:** 10.1007/s10936-025-10170-4

**Published:** 2025-08-20

**Authors:** Perry Susskind

**Affiliations:** Department of Mathematics & Statistics, Connecticut College, 270 Mohegan Avenue, New London, CT 06320 USA

**Correction to: Journal of Psycholinguistic Research (2025) 54:47**

10.1007/s10936-025-10158-0

In the section “Derived Measures” the 3rd parenthetical remark read as “(For our sample text we have, L = 5, H = 12, N = 17.)” but should have been written as “(For our sample text we have, |L| = 5, |H| = 12, N = 17.)”.

The original article has been corrected.

